# Effectiveness of Curing Compounds for Concrete

**DOI:** 10.3390/ma15072699

**Published:** 2022-04-06

**Authors:** Filip Chyliński, Agnieszka Michalik, Mateusz Kozicki

**Affiliations:** Instytut Techniki Budowlanej, 00-611 Warsaw, Poland; a.michalik@itb.pl (A.M.); m.kozicki@itb.pl (M.K.)

**Keywords:** concrete, concrete care, curing compound, curing efficiency index, water retention

## Abstract

Curing compounds are widely used materials that are used in place of other methods of curing fresh concrete. The article presents an overview of the effectiveness of the concrete curing compounds widely used in Europe. Eleven different products have been tested. FTIR spectroscopy identification tests showed that all tested products might be divided into two main groups, depending on the type of their active substance. The water retention efficiency of each curing compound was examined, and the tensile strength of the cured samples was tested using the pull-off method. The dry mass content of the tested products was examined to check for a correlation between their effectiveness and active substance content. The microstructure of mortars treated with the most effective compounds and the reference mortar were examined using SEM techniques. Significant differences in microstructure were found between cured samples with different curing compounds, and also with uncured samples.

## 1. Introduction

The durability of concrete defines its ability to stand against an aggressive environment with which it has contact [1,2]. This is even more important in sustainable development, where an extended service life is favourable [3]. Curing is one of the most important tasks that must be performed after properly placing the concrete in the building object. The European standard EN 13670 and the American Concrete Institute (ACI) recommend that the concrete in its early life, after placing, compacting and surface finishing, shall be cured to minimise plastic shrinkage, ensure adequate surface strength, and ensure adequate surface zone durability [4,5,6,7,8]. It should also be protected from harmful weather conditions, freezing, harmful vibration, impact, or damage [9]. Currently, insufficient curing is still a common cause of defects in concrete structures. The role of proper curing of young concrete is the subject of many publications [10,11,12,13,14]. Even the best-designed concrete containing the appropriate components will not achieve its properties without proper placing of the concrete mix (including compaction) and curing of the fresh concrete. The curing methods should achieve low evaporation rates from the concrete’s surface or keep the surface permanently wet. In some cases, natural curing might be sufficient, e.g., when the weather is misty, and the air humidity is high enough to protect it from excessive water evaporation or dampness. The generally accepted definitions of concrete curing define it as treatments undertaken from the moment of placing and compacting the concrete mix, aimed at ensuring the proper course of cement hydration processes and, as a result, obtaining concrete with the required designed properties within a specified time [4,12]. In order to ensure the required durability of concrete, the key is both the proper selection of the curing method and its duration [4,5,7]. A lack of maintenance or improper implementation is often the cause of damage and/or defects to the structure, which are then the subject of a dispute between the parties to the investment. There are two main methods of concrete moisture care: wet and coating. Wet maintenance consists in bringing water to the surface of the concrete, which the concrete can absorb. This requires that the concrete surface be continuously in contact with the water for a certain period of time. Such conditions can be achieved by continuously sprinkling or pouring water on the concrete, or by laying a soaked cloth onto the concrete. This method does not always provide good results, e.g., when caring for vertical surfaces or uneven areas, and in windy and sunny weather. Moreover, maintaining the water saturation of very large concrete surfaces, such as floors or road surfaces, is often impossible. Concrete curing methods based on reducing the evaporation of water from the concrete’s surface without adding excessive water from the outside might be carried by using sheets and rolls of materials (e.g., plastic film, paper reinforced with a bituminous binder) or by using liquid film-forming preparations, which are the most popular techniques of concrete care, apart from sprinkling water. The method consists of spraying a curing compound that forms a coating which protects the concrete against excessive water evaporation. Currently, the most commonly used active substances are wax and paraffin emulsions and synthetic resin solutions [11,15]. Paraffin and wax emulsions are difficult to remove after the finished curing process, leaving a slippery surface, which has to be extensively brushed before further work can be performed. These preparations are most often water dispersions of a white colour, which enables an even application of the preparation on the surface and thus obtains a homogeneous, tight coating. The coating application must be continuous and undamaged. The spray time is also important. The preparation should be applied immediately after confirming the absence of water on the concrete surface, but before it is completely dry; that is, when the surface becomes matte [16,17,18]. Preparations can be applied by spraying or painting. This method of curing is popular in regions where the climate is characterised by high variability of weather conditions, temperature, air humidity and wind intensity in a short period of time, which might disrupt the cement hydration process in the concrete and affects its durability. Also, selection of the appropriate curing compound and duration of curing is important. When choosing a curing agent, apart from the economic issue, its effectiveness depends on the chemical composition, the method of application and its impact on the surface layer of concrete.

There are no global regulations and requirements for curing compounds, and each country or region mostly deals on its own. The standard that contains requirements for such products is ASTM C 309, which is not valid in Europe [19]. According to the Construction Product Directive (CPR), in European countries curing compounds are not a construction product because these substances are not permanently built in the building object [19]. For this reason, there is no reference document containing a set of test methods and requirements for this type of product. However, their production and use are widespread. There is a Polish national document, which in fact is not a standard but a recommendation, although it contains some requirements for these products [20].

This paper aimed to show the differences between the properties of the most popular and widely used curing compounds in Europe. Their effectiveness was determined by testing water retention efficiency. Because manufacturers declare similar types of active substances, the FTIR spectrum (Nicolet iS10, Thermo Scientific, Waltham, MA, USA) of curing compounds was performed to check their similarity. Due to the fact that efficiency of the curing compound might also be caused by the active substance’s content, the dry mass content was also determined. Considering that the curing process might affect the tensile strength of cement composites, especially in regions near the surface, the tensile strength of cured and uncured samples was tested using the pull-off method. Also, the microstructure of cured and uncured cement composites was examined using scanning electron microscopy (SEM, Carl Zeiss Microscopy GmbH, Köln, Germany) techniques to discover the differences between the formed structures.

The novelty of this paper is that the presented results of these tests compare the properties of products available in the market and show that a curing compound’s effectiveness might vary a great deal from product to product.

## 2. Materials and Methods

The materials used for tests were 11 types of curing compounds available in the market and widely used on construction sites. The names of products and companies were coded to avoid any conflicts of interest, and the samples were randomly numbered from 1 to 11. Each compound was used exactly following the directions given by the manufacturer.

### 2.1. FTIR Spectroscopy

**Figure 1:**
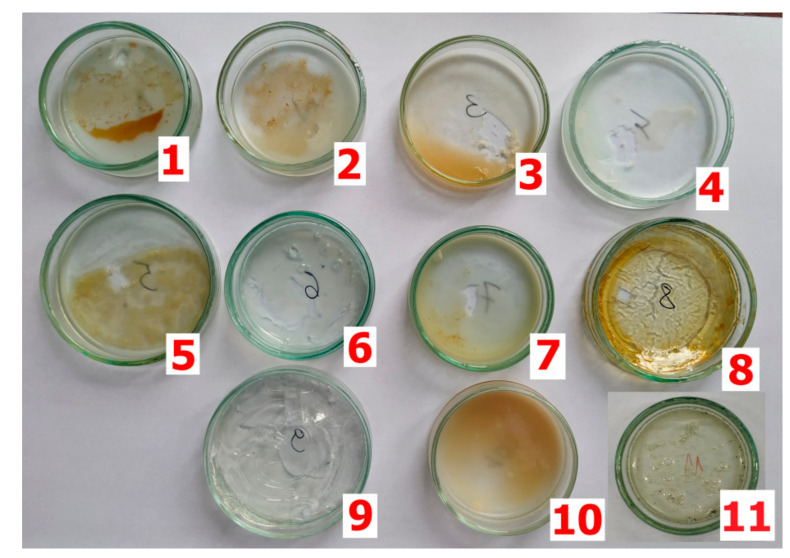
Samples of curing compound after drying (No. 1–11).

Curing compound samples were dried in an oven at 105 °C for 24 h (Figure 1). After cooling, the FTIR spectra of the curing compound were determined by the Attenuated Total Reflectance (ATR) mode using an FTIR spectrometer (Nicolet iS10, Thermo Scientific, Waltham, MA, USA), over a scan range of 500 to 4000 cm^−1^, using 16 scans. The spectral resolution was equal to 4 cm^−1^. Thermo Scientific OMNIC software (version 9, software version number: 9.2.41, Thermo Fisher Scientific OMNIC, Waltham, MA, USA) was used to process the collected data. In addition to the commonly used transmission measurements, in which the infrared radiation beam is weakened when it passes through the sample absorbing radiation, reflection techniques are also used. In these techniques, radiation undergoes multiple internal reflections in the crystal of high refractive index (usually made of Ge, ZnSe or diamond). In the case of a liquid sample, pouring a shallow amount over the surface of the crystal is sufficient [21]. A system constructed in this way is called an ATR system (attenuated total reflection) or the method of attenuated total internal reflection.

### 2.2. Dry Mass Content

The dry mass content in curing compounds was tested according to the EN 480-8 standard [22]. Samples were dried in an oven at 105 °C until they reached a constant mass. These tests aimed to check if the compounds’ effectiveness is related to the dry mass content.

### 2.3. Water Retention Efficiency and Effectiveness of Curing Compounds

The effectiveness of curing compounds is defined as their ability to prevent the evaporation of water from fresh concrete under defined temperature and humidity conditions. The most widely used methods are PKN-CEN/TS 14754-1, similar to ASTM C156 and AASHTO T 155 [17,18,23]. The differences between the test methods are described by Vandenbossche [24]. This paper tested eleven curing compounds using the European Technical Specification PKN-CEN/TS 14754-1 [16]. According to this document the effectiveness of curing compounds should be tested on referenced concrete containing cement (acc. to EN 197-1 [25]), aggregates and additives (acc. to EN 12620 [26]) and water in mass ratio 1.0: 3.0: 0.42. The consistency of concrete mix should be S1 slump loss according to EN 206 and measured according to EN 12350-2 [27,28]. Maximum air content measured according to EN 12350-7 should not exceed 3% [29]. Melaphyre fines were added to decrease the bleeding effect, which makes it difficult to determine the moment of application of the curing compound. The composition of the tested concrete is presented in Table 1.

After mixing, the concrete was placed in moulds with a surface of 50,000 ± 5000 mm^2^ and a depth of 50 ± 2 mm. After moulding and finishing the surface samples, they were left for approximately an hour to evaporate. Then, the curing compounds were sprayed over the surface in two cross layers, in the quantities required by their manufacturers. Each curing compound was tested on three samples. Also, a reference sample without curing compounds was tested. The samples were than placed in a climate chamber with a temperature of 35 ± 2 °C and a relative humidity of 40 ± 3% with no air circulation on the top of the specimens. Each sample was weighed before placing in the chamber and after 6 h, 24 h and 72 h in the climate chamber. Also, the loss of solvent for each curing compound was determined using a nonadsorbing plate with the same surface as the tested samples containing the same amount of curing compound. The solvent loss was determined by weighing the plates cured in the same conditions in the climate chamber after the same amount of time as the tested concrete samples. Water retention efficiency is expressed by the curing efficiency index *I*_t,_ which is calculated for the curing compound at the time *t* as a percentage from the Equation (1).

(1) $$I_{t}=\frac{M_{Rt}-M_{Ct}}{M_{Rt}}\times 100$$

where:

*I_t_*—is the curing efficiency index after time “*t*”
*T*—is the time at which the weight of the specimens is determined (*t* = 0 h, 6 h, 24 h and 72 h)
*M_Rt_*—is the average of mass loss from the untreated specimens at time “*t*” (specimens without curing compound)
*M_Ct_*—is the average of mass loss from the treated specimens at time “*t*” reduced with the average of mass loss of the curing compound (solvent loss) at time “*t*”.

### 2.4. Tensile Strength by the Pull-Off Method

The tensile strength tests aimed to discover any differences between the cured and uncured samples as insufficient curing might decrease the tensile strength of the concrete near the cured surface. Tests were carried out on concrete samples with the composition given in Table 1. After placing fresh concrete in moulds (300 mm × 300 mm × 50 mm) and trowelling, the surface curing compounds were sprayed over the surface. For the test, the curing compounds with No. 1–4, 7, 9, 10 were chosen, excluding No. 5, 6, 8 and 11 as those characterised with the lowest values of curing efficiency index. Also, a reference sample with no curing was prepared. The samples were kept in moulds for 28 days under laboratory conditions (temperature 20 ± 2 °C and humidity 60 ± 5%). After removing the residues of curing compounds by brushing, the tensile strength was determined using the pull-off method according to EN 1542 by using the Proceq equipment model DY-216 with 50 mm discs [30]. The loading rate was 0.05 ± 0.01 MPa/s. The discs were stuck directly on the surface of the concrete using epoxy resin after drilling the surface of the concrete using a core drill. The tensile strength was measured five times for each sample, and the average values were calculated.

### 2.5. Scanning Microscopy

Structure observations were made using a scanning electron microscope (SEM) produced by Zeiss, model Sigma 500 VP (Carl Zeiss Microscopy GmbH, Köln, Germany). Secondary electron (SE) and backscattered electron (BSE,) images were collected. Phase compositions and mapping were analysed using the energy-dispersive X-ray spectroscopy (EDS) detector model Oxford Ultim Max 40 (Oxford Instruments, High Wycombe, UK).

**Figure 2:**
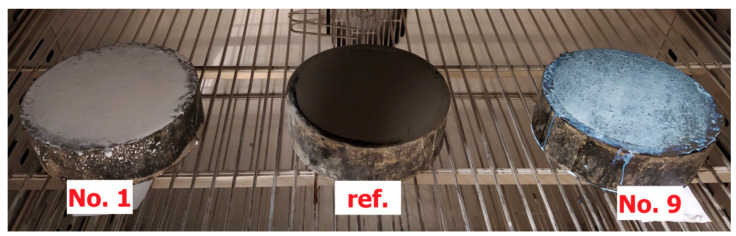
Samples of mortar cured in the oven (reference sample and No. 1 and 9).

According to EN 196-1 [31], samples of fresh standard mortar were treated with two curing compounds, No. 1 and No. 9—one for each type of active substance and with the highest dry mass content. Also, a reference mortar was prepared without any surface curing. The samples were kept in cylindrical moulds with a diameter of 160 mm and a height of 50 mm until the 7th day in an oven at a temperature of 40 °C with air circulation (Figure 2). Such conditions are also allowed by the PKN-CEN/TS 14754-1 in water retention efficiency tests [16].

After demoulding, a 5 mm slice of mortar was cut through the centre of each sample, perpendicular to the trowelled surface. Then, a smaller piece was cut (50 mm × 20 mm × 5 mm) for microscopic examinations. Next, these pieces were dried in an oven at a temperature of 40 °C and put into epoxy resin under a vacuum for better filling of the air voids. The final step of preparing the samples was polishing their surface, and the samples were then gold evaporated before examining them under a microscope. Structural observations were only examined for one of each type of active substance in the curing compound (with the highest dry mass content) and the reference mortar without any surface curing.

## 3. Results and Discussion

### 3.1. FTIR Spectroscopy

**Figure 3:**
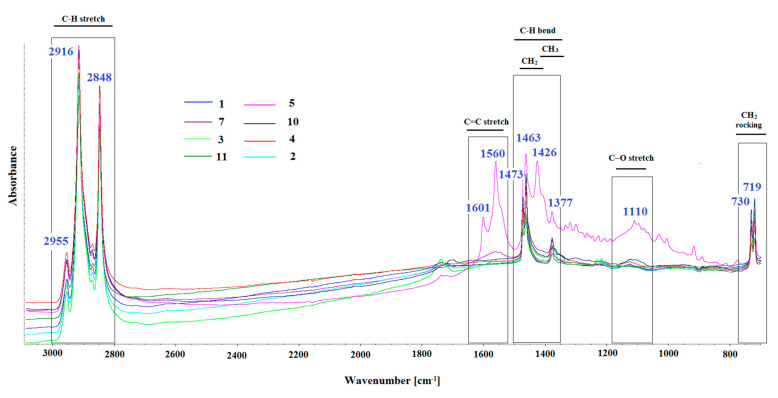
FTIR spectra of curing compound for concrete for samples no. 1–5, 7 and 10–11.

Figure 2 and Figure 3 present the FTIR spectra of curing compounds marked with numbers 1-11 (Figure 1). It was decided to divide them into two groups because the correlation between the spectra from the two groups averaged 15–30%. Both figures include spectra with a correlation of more than 85% for each group.

Figure 3 shows the FTIR spectra of curing compound for concrete for samples No. 1–5, 7 and 10–11. All of these spectra are notably similar to each other. The FTIR spectra of C-H stretching vibrations of saturated hydrocarbons are seen below 3000 cm^−1^ (in a range of 2800–3000 cm^−1^), -CH_3_ and -CH_2_ deformations at about 1463 cm^−1^ and 1377 cm^−1^. Moreover, sample No. 5 gives additional bands, and is apparently doped with some other substances. The band at 1110 cm^−1^ is identified as the C–O bond stretching, and the bands at 1560 and 1601 cm^−1^ can be assigned as C=C stretching vibrations. Rocking and wagging of -CH_2_ give clear twin bands at 719 cm^−1^ and 730 cm^−1^. This pair of peaks indicates crystallinity in the sample. In molten or disordered samples, a single peak would appear at approximately 725 cm^−1^, whereas in crystalline samples, this peak splits into twin peaks [32,33].

**Figure 4:**
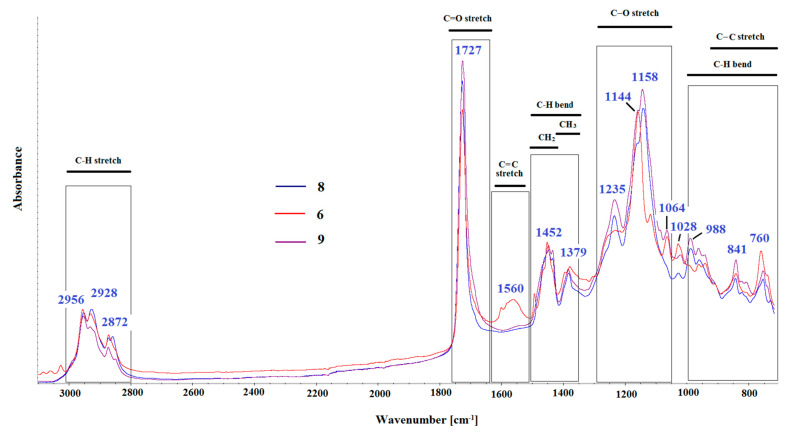
FTIR spectra of curing compound for concrete for samples No. 6 and 8–9.

The spectra presented in Figure 4 show a completely different distribution of the bands. A sharp, intense peak at 1727 cm^−1^ appeared due to the presence of ester carbonyl group C=O stretching vibration. The peaks ranging from 1250–1150 cm^−1^ can be explained as owing to the C-O (ester bond) stretching vibrations. The bands at 1452 cm^−1^, 1379 cm^−1^ and 760 cm^−1^ can be attributed to the bending vibrations of the C–H bonds. The bands at 841 cm^−1^, 988 cm^−1^ and 1064 cm^−1^ are the characteristic absorption vibration of poly (methyl methacrylate) or similar polymer systems [34]. The three bands at 2956 cm^−1^, 2928 cm^−1^ and 2872 cm^−1^ can be assigned to the C–H bond stretching vibrations of the –CH_3_ and –CH_2_ groups. Spectrum No. 6 probably shows some organic modifier added to the curing compound for concrete in the presence of the band at 1560 cm^−1^, which can be assigned as C=C stretching vibration.

Based on the FTIR results for all the tested curing compounds for concrete, it can be concluded that they belong to two different groups. It can be assumed that the raw material base for products. No. 1–5, 7 and 10–11 are paraffins. The mutual correlations of the spectra to each other are above 95%. Product No. 5 has a slightly different spectrum (a modified composition), but still, the match with the rest of the group is high and amounts to over 85%.

FTIR spectra show the raw material base for products. No. 8 and 9 belong to the second group of products, modified synthetic resins (acrylic). The mutual correlations of spectra No. 8 and 9 are very high, and amount to over 95%. The match between spectrum No. 6 and the other spectra is over 85%, which proves the modification of the polymer composition.

### 3.2. Dry Mass Content

Table 2 presents the dry mass content of tested products. The content of dry mass in products containing paraffin as an active substance (No. 1–4, 7, 10, 11) varies substantially between 6.2% and 27.3%. In product No. 5, where paraffin is the main active substance, the dry mass content is lower than 1.5%. This might be caused by the evaporation of other active substances during drying. The dry mass of samples No. 8 and No. 9 containing modified synthetic resins was 14.4% and 26.3%, respectively. In product No. 6, where the active substance was slightly different from No. 8 and No. 9, the dry mass content was 9.1%.

### 3.3. Water Retention Efficiency and Effectiveness of Curing Compounds

**Figure 5:**
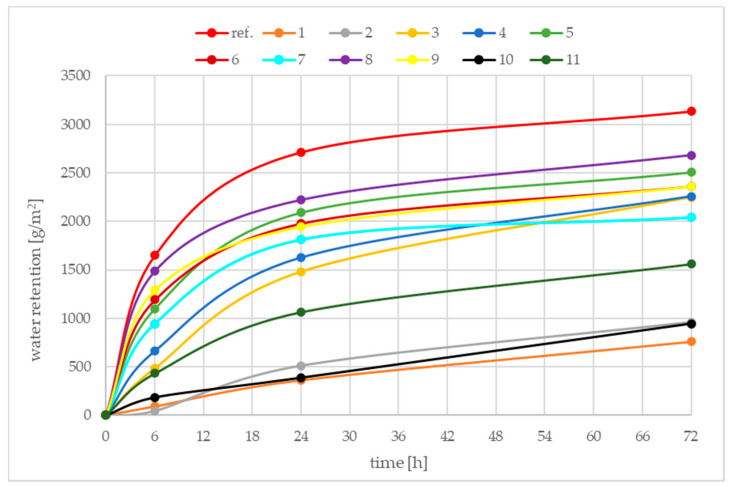
Water retention for all examined curing compounds samples over 72 h.

Figure 5 presents the results of water retention efficiency tests. Curves represents the quantity of evaporated water during the time. The highest quantities of water evaporated from the sample of concrete untreated with any curing compound. The lower the quantity of water evaporated, the more effective the curing compound is.

Analysing the results of tests, compound No. 2 has the lowest water retention value after 6 h, meaning that it prevents the most water evaporation in this period of time, out of all the tested compounds. After 24 h, the most effective curing might be obtained using compound No. 1 or No. 10. The most effective curing compound after 72 h is No. 1, and No. 2 and No. 10 are a little less effective.

The requirements of the ASTM C 309 standard for water retention efficiency after 72 h is not more than 0.55 kg/m^2^ [18]. When comparing the received test values to those requirements, a conclusion might be derived from the fact that none of the tested compounds fulfil this requirement. However, the testing method according to ASTM differs from the European test method, e.g., lower temperature during the test (23 ± 2 °C) and higher humidity (50 ± 10%). So, the received values cannot be compared with those requirements.

**Figure 6:**
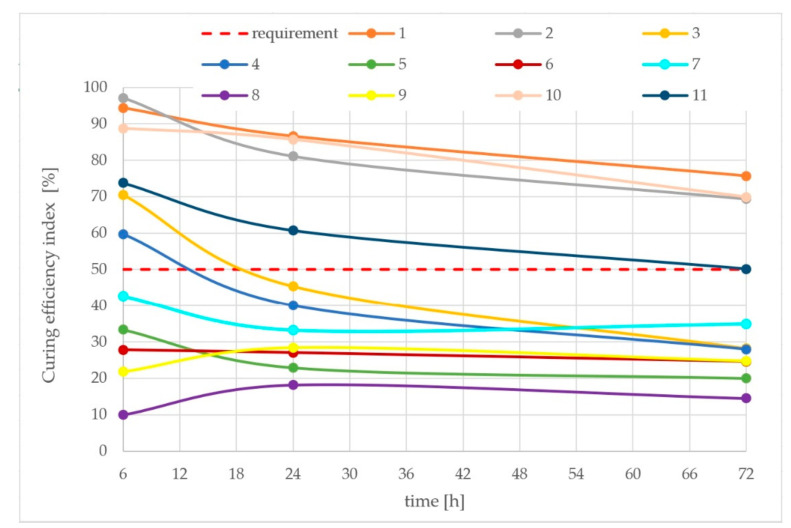
Curing efficiency index for all examined curing compounds samples during 72 h.

Figure 6 presents the results of calculated values of the curing efficiency index. Curves represents the efficiency of curing compounds. The lower values a curing compound reaches, the more effective it is in the curing of concrete.

According to the Polish national recommendation curing efficiency requirements, the index should be not less than 50% after 3 days of curing [20]. Curing compounds No. 1, 2, 10 and 11 fulfil these requirements, but others do not. According to the received results of tests, those compounds will not fulfil the Polish national recommendation requirements.

**Figure 7:**
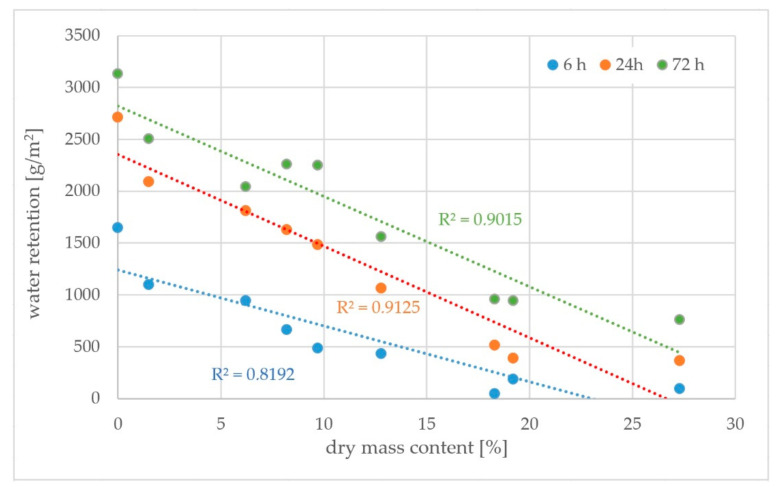
Correlation between water retention and dry mass for paraffin curing compounds.

**Figure 8:**
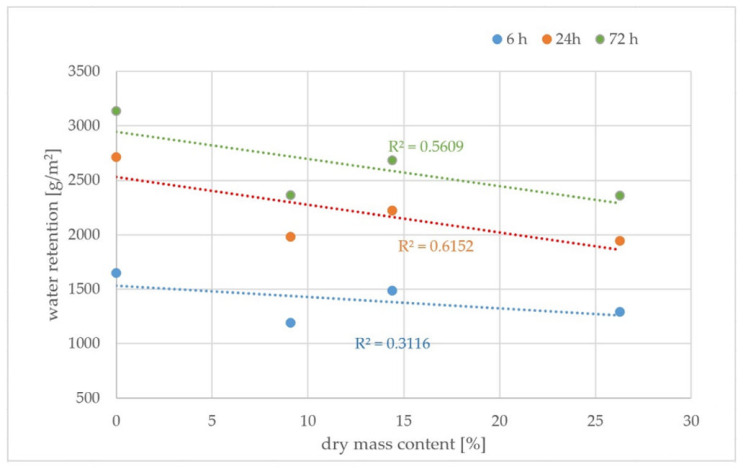
Correlation between water retention and dry mass for modified synthetic resins curing compounds.

Due to the fact that the effectiveness of curing compound is related to the content of the active substance in the product, the correlation between water retention and dry mass content has been calculated. Figure 7 and Figure 8 present a correlation between water retention after a different amount of time and the content of dry mass—paraffin or modified synthetic resins, respectively.

Analysis of correlations in Figure 6 where the received values of R^2^ are in a range of 0.82–0.91 and shows that the effectiveness of water retention in curing compound is highly related to the content of paraffin in it—the higher is the content of paraffin, the better it prevents water evaporation. This might suggest that the mechanism of preventing water evaporation is related only to the ability of the compound to close the surface of fresh concrete with a sealed layer.

The correlation between water retention and dry mass content for curing compounds containing modified synthetic resins is relatively low, and the R^2^ values are 0.31, 0.62 and 0.56 for 6, 24 and 72 h of curing, respectively. This shows that the mechanism of curing fresh concrete using those curing compounds might be more complex than in paraffin compounds, and the effectiveness is related not only to the thickness of curing resin film but also to other aspects such as depth of penetration into the concrete or varying content of volatile active substances that evaporate at 105 °C.

### 3.4. Tensile Strength by the Pull-Off Method

Table 3 presents the results of tensile strength determined by the pull-off method.

The tensile strength of the surface area of concrete for all tested products was greater than for the reference sample. The highest average value was found in samples No. 1 and No. 2, which also had the best water retention efficiency. This shows that effectively curing the surface of concrete and preventing excessive water evaporation allows concrete to reach higher tensile strength values.

### 3.5. Scanning Microscopy

**Figure 9:**
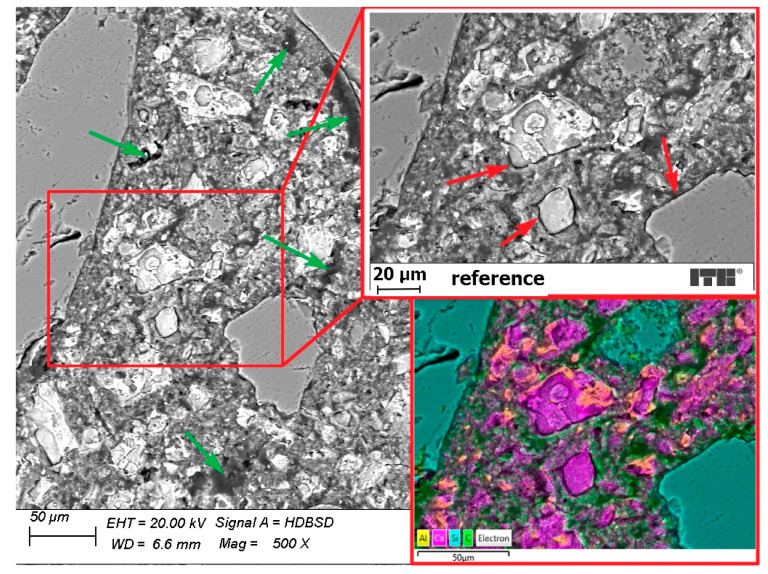
Reference mortar without curing (red arrows—cracks, green arrows—air voids).

Figure 9 presents BSE (backscattered electron) images and an EDS mapping (energy-dispersive X-ray spectroscopy) of reference mortar without any curing. The mortar structure was full of cracks (red arrows) and air voids (green arrows). The C-S-H phase was not well-formed and weakly bonded to the clinker and aggregate grains as the cracks occurred in the transition zone. Numerous air voids show that the hydration process was slowed down or even disrupted after some time. Air voids are also visible on the EDX mapping image as a green area filled with resin, due to sample preparation.

**Figure 10:**
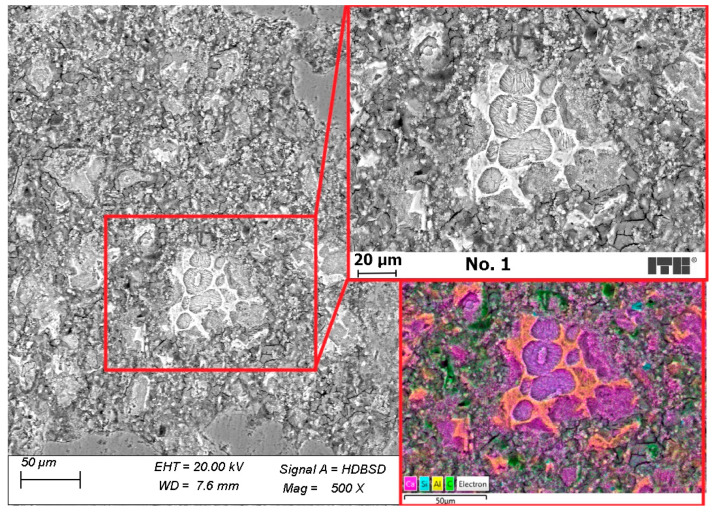
Mortar cured with product No. 1.

Figure 10 presents BSE and EDX-mapping images of a sample of mortar cured with preparation No. 1, containing paraffin-water dispersion. Compared to the reference mortar, the microstructure of mortar was well-formed, and the C-S-H was compact. The transition zone between C-S-H gel and clinker grains and aggregate grains was sealed and well bonded. A few cracks might occur due to the sample preparation, especially during the drying process. The green areas on the EDX-mapping image representing air voids filled with resin were much smaller than those in the reference sample.

**Figure 11:**
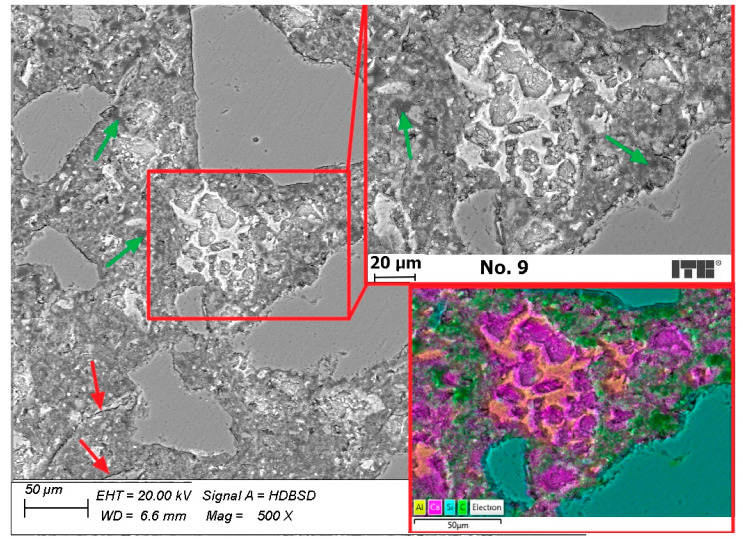
Mortar cured with product No. 9 (red arrows—cracks, green arrows—air voids).

The microstructure of the mortar cured with preparation No. 9, containing polymer dispersions, is presented in Figure 10. It is similar to the structure of the mortar cured with preparation No. 1 and far better developed than the microstructure of the reference sample (Figure 9). The number of air voids and cracks in the microstructure of the mortar cured with No. 9 was greater than in the mortar cured with No. 1. The air voids are also visible on EDX-mapping (Figure 11) as a green area filled with resin.

Analysing the microstructure of mortars using SEM techniques, a conclusion might be drawn that curing concretes with curing compounds containing paraffin is effective and leads to properly forming the microstructure of cement composites due to the prevention of water evaporation from the structure. Effectiveness of curing compounds containing modified synthetic resins as an active substance is lower than those with paraffin because numerous air voids and some cracks were also observed in the structure, although curing compounds containing polymers are much more effective in preventing the structure from drying than not using any curing compounds.

## 4. Conclusions

Despite the importance of the curing process and its impact on the durability of concrete objects being common knowledge, there are still cases of neglect in this regard, which result in various types of structural destruction. As shown in this study, curing concrete with the use of curing compounds might be effective in protecting the concrete against excessive drying, which serves to improve concrete properties compared with uncured concrete.

By analysing the results of tests presented in this paper and comparing them with the literature data and standards, the following conclusions might be driven:

- Based on the functional groups’ vibrations (FTIR), it can be concluded that all tested curing compounds for concrete belong to two different groups. Both groups have different characteristic vibrations, while the fingerprint regions give unique peaks of configuration which can be used to distinguish between them. The vibrations present in the first group (samples No. 1–5, 7 and 10–11) indicate that the curing compounds are based on paraffins, while the vibrations in the second group indicate modified synthetic resins.
- Water retention and curing efficiency index for products available on the market have a wide range of values. Due to the lack of standardised test methods and requirements in European countries, the obtained values should be analysed at the place of use. It should be assumed that all of them might be used for the curing of concrete.
- Water retention efficiency correlates well with the dry mass content in curing compounds containing paraffin. That might show that the main mechanism of curing this type of product is preventing water evaporation from the concrete by creating a sealed barrier.
- Effectively cured concrete using curing compounds with low values of curing efficiency index is characterised by higher tensile strength on its surface area. That proves the importance of curing the concrete to reach projected values of performance properties.
- The effectiveness of curing compounds affects the microstructure of cement composite. When the microstructure is compact and tight, and the C-S-H phase is well developed, the effectivity of the curing compound is high. Conversely, when using low effectiveness curing compounds or none at all, the structure has numerous air voids and cracks and the C-S-H phase is weakly developed.

These test results of commonly used concrete curing compounds can have an important practical impact. Research have shown that in the process of concrete care, the chemical composition (type of base) and the dry mass content of the curing compound is important. Properly selected and applied curing compound contributes to an increase of strength properties and the improvement of the microstructure of the surface and subsurface concrete layers.

## Figures and Tables

**Table 1:** Composition of tested concrete.

Constituent	Content (kg/m^3^)
Portland cement CEM I 42,5 R	520
melaphyre fines 0–0.25	182
fine aggregate–river sand 0/2 mm	589
coarse aggregate–natural 2/8 mm	846
tap water	220
water–cement ratio	w/c = 0.42
consistency of the fresh mix	S1

**Table 2:** Dry mass content in tested products.

Sample	Dry MASS Content (%)
1	27.3
2	18.3
3	9.7
4	8.2
5	1.5
6	9.1
7	6.2
8	14.4
9	26.3
10	19.2
11	12.8

**Table 3:** Tensile strength by the pull-off method.

Sample	Tensile Strength (Standard Deviation) (MPa)	Minimum Value (MPa)
ref.	1.82 (0.16)	1.56
1	2.63 (0.30)	2.22
2	2.53 (0.28)	2.26
3	1.95 (0.19)	1.70
4	2.51 (0.12)	2.37
7	1.87 (0.16)	1.67
9	2.11 (0.17)	2.03
10	1.87 (0.17)	1.80
